# Genome-Wide Identification of R2R3-MYB Transcription Factors: Discovery of a “Dual-Function” Regulator of Gypenoside and Flavonol Biosynthesis in *Gynostemma pentaphyllum*

**DOI:** 10.3389/fpls.2021.796248

**Published:** 2022-01-05

**Authors:** Ding Huang, Ruhong Ming, Shiqiang Xu, Shaochang Yao, Liangbo Li, Rongshao Huang, Yong Tan

**Affiliations:** ^1^College of Pharmacy, Guangxi University of Chinese Medicine, Nanning, China; ^2^Guangxi Key Laboratory of Zhuang and Yao Ethnic Medicine, Guangxi University of Chinese Medicine, Nanning, China; ^3^Guangdong Provincial Engineering and Technology Research Center for Conservation and Utilization of the Genuine Southern Medicinal Resources, Guangzhou, China; ^4^Guangdong Provincial Key Laboratory of Crops Genetics and Improvement, Crops Research Institute, Guangdong Academy of Agricultural Sciences, Guangzhou, China

**Keywords:** *R2R3-MYB* gene family, *Gynostemma pentaphyllum*, gypenoside, flavonol, transcription factor

## Abstract

The *R2R3-MYB* gene family participates in several plant physiological processes, especially the regulation of the biosynthesis of secondary metabolites. However, little is known about the functions of *R2R3-MYB* genes in *Gynostemma pentaphyllum* (*G. pentaphyllum*), a traditional Chinese medicinal herb that is an excellent source of gypenosides (a class of triterpenoid saponins) and flavonoids. In this study, a systematic genome-wide analysis of the *R2R3-MYB* gene family was performed using the recently sequenced *G. pentaphyllum* genome. In total, 87 *R2R3-GpMYB* genes were identified and subsequently divided into 32 subgroups based on phylogenetic analysis. The analysis was based on conserved exon–intron structures and motif compositions within the same subgroup. Collinearity analysis demonstrated that segmental duplication events were majorly responsible for the expansion of the *R2R3-GpMYB* gene family, and Ka/Ks analysis indicated that the majority of the duplicated *R2R3-GpMYB* genes underwent purifying selection. A combination of transcriptome analysis and quantitative reverse transcriptase-PCR (qRT-PCR) confirmed that *Gynostemma pentaphyllum* myeloblastosis 81 (*GpMYB81*) along with genes encoding gypenoside and flavonol biosynthetic enzymes exhibited similar expression patterns in different tissues and responses to methyl jasmonate (MeJA). Moreover, GpMYB81 could bind to the promoters of *Gynostemma pentaphyllum* farnesyl pyrophosphate synthase 1 (*GpFPS1*) and *Gynostemma pentaphyllum* chalcone synthase (*GpCHS*), the key structural genes of gypenoside and flavonol biosynthesis, respectively, and activate their expression. Altogether, this study highlights a novel transcriptional regulatory mechanism that suggests that GpMYB81 acts as a “dual-function” regulator of gypenoside and flavonol biosynthesis in *G. pentaphyllum*.

## Introduction

The myeloblastosis (MYB) transcriptional regulators comprise one of the largest and important families in the plant kingdom (Riechmann et al., 2000). A classical characteristic of MYB proteins is that they contain conserved MYB DNA-binding domain repeats in the N-terminus. The conserved MYB domain is composed of one to four continuous and non-redundant imperfect sequence repeats, designated as R1, R2, and R3 according to their similarity to the c-MYB protein. Each conserved MYB DNA-binding domain repeat is approximately 50 amino acids in length and encodes three α-helices (Dubos et al., 2010). Depending on the number of MYB repeats in the MYB domain, MYB transcription factors (TFs) can be subdivided into R2R3-MYB (2R-MYB), R1R2R3-MYB (3R-MYB), 4R-MYB (containing four R1/R2 repeats), and the MYB-related subfamily (containing a single or a partial MYB repeat) (Stracke et al., 2001). In the R2R3-MYB family, the last two α-helices of each MYB repeat form a helix–turn–helix (HTH) structure and the third α-helix of R2 and R3 repeats are essential for DNA binding, allowing direct contact and insertion into the DNA major groove (Gabrielsen et al., 1991; Jia et al., 2004).

The R2R3-MYB TFs constitute the largest MYB subfamily in plants and the majority of them can specifically recognize the MYB-core sequence [(C/T)NGTT(G/T)] and AC-rich element [A/CCC(T/A)A(C/A)C/G] (Zhu et al., 2015; Millard et al., 2019). As important regulatory proteins involved in several crucial biological processes, the number of identified and characterized R2R3-MYB TFs in plants is continuously increasing. R2R3-MYB TFs play vital roles in plant growth and development, respond to various biotic and abiotic stresses, and regulate secondary metabolism, especially those affecting nutrition and medicinal components or appearance and quality traits (Huang D. et al., 2019; Wang et al., 2019; He et al., 2020). For example, *Ruby1* and *Ruby2* encode R2R3-MYB TFs and form a gene cluster that shows a regulatory subfunctionalization in anthocyanin biosynthesis in citrus (Huang et al., 2018). Similarly, kiwifruit R2R3-MYB TF MYB7 acts as a positive regulator to activate the promoter of the key gene lycopene beta-cyclase (*AdLCY-b*) in the carotenoid biosynthetic pathway (Ampomah-Dwamena et al., 2019). The genes *AtMYB11*, *AtMYB12*, and *AtMYB111* from subgroup 7 of the *Arabidopsis thaliana* (*A. thaliana*) *R2R3-MYB* gene family control flavonol accumulation in different parts of the *A. thaliana* seedling (Stracke et al., 2007).

Extensive study on the *R2R3-MYB* gene family members in horticultural and crop plants has increased our understanding of their functions and transcriptional regulatory mechanism. However, characteristics of this gene family in *Gynostemma pentaphyllum* (*G. pentaphyllum*), a traditional Chinese medicinal herb named jiaogulan, have not yet been declassified. As an economically valuable medicinal and edible plant, jiaogulan tea has been commercialized globally. Gypenosides are a major class of triterpenoids with a dammarane-type carbon skeleton in *G. pentaphyllum*, which exert beneficial effects on human health (Park et al., 2014; Shen et al., 2020; Wang et al., 2020). In addition, flavonoids can be divided into flavonols, flavones, flavonones, and anthocyanidins that play important roles in medicine and hygiene due to their high antioxidant activity (Chen and Chen, 2013; Wang et al., 2018). Gypenosides are the primary active components of *G. pentaphyllum*. In contrast, flavonols extracted from *G. pentaphyllum* contain mainly kaempferol and quercetin derivatives, which were considered major contributors to the beneficial properties of *G. pentaphyllum* (Xie et al., 2011). In a recent study, gypenoside biosynthetic genes, including *farnesyl pyrophosphate synthase* (*FPS*), *squalene synthase* (*SS*), *squalene epoxidase* (*SE*), *2,3-oxidosqualene cyclase* (*OSC*), and *cytochrome P450* (*CYP450*), have been well elucidated (Huang et al., 2021). In addition, structural genes of the flavonoid metabolic pathway are best understood at present (Nabavi et al., 2020). However, it remains poorly understood whether the *R2R3-MYB* gene family members are involved in the regulation of both gypenoside and flavonol biosynthesis in *G. pentaphyllum*.

The recently published *G. pentaphyllum* genome sequence provides a convenient tool to identify and characterize the *R2R3-GpMYB* gene family (Huang et al., 2021). In this study, we performed a genome-wide identification of *R2R3-MYB* genes in *G. pentaphyllum* and screened 87 *R2R3-GpMYB* genes. Next, a comprehensive analysis including phylogenetic relationship, gene structure, conserved domains and motifs, chromosomal location, gene duplication, and collinearity was performed. Based on the weighted gene co-expression network analysis (WGCNA) and expression pattern response to methyl jasmonate (MeJA) treatment, GpMYB81 was suggested as a “dual-function” TF that can regulate both gypenoside and flavonol biosynthesis. In addition, GpMYB81 could bind to the promoters of *GpFPS1* and *GpCHS* genes and activate their transcription, thus opening up the possibility for improving the yield of both gypenosides and flavonols in *G. pentaphyllum* through metabolic engineering.

## Materials and Methods

### Plant Materials and Methyl Jasmonate Treatment

Plant materials were cultivated in a fully controlled climate room of Guangxi University of Chinese Medicine (Nanning, China), with a 16-h light/8-h dark cycle at 24°C temperature. *G. pentaphyllum* seedlings were culture in Hoagland’s nutrient solutions. For MeJA treatment, 6-week-old *G. pentaphyllum* seedlings were cultured in Hoagland’s nutrient solutions with 100 μm MeJA. For quantification of gene expression using quantitative reverse transcriptase-PCR (qRT-PCR), *G. pentaphyllum* seedlings were collected at 0, 6, 12, and 24 h after MeJA treatment; the leaves of three seedlings were randomly selected to form three biological replicates. All the plant samples were frozen with liquid nitrogen and stored at −80°C.

### Identification of *Gynostemma pentaphyllum R2R3-MYB* Family Genes

The Hidden Markov Model (HMM) file of MYB DNA-binding domain (PF00249), obtained from the Pfam database,^1^ was used as the query for HMM search using HMMER 3.0 (Finn et al., 2011) to identify *MYB* genes from *G. pentaphyllum* genome with default parameters. To ensure the presence of two MYB DNA-binding domain repeats, candidate MYB protein sequences were further examined using the Simple Modular Architecture Research Tool (SMART) database.^2^ Finally, a manual inspection was performed to confirm the reliability of our results.

### Sequence Analysis and Phylogenetic Analysis of *R2R3-MYB* Genes

The exon/intron structure of all the *R2R3-GpMYB* genes was displayed using the TBtools software (Chen et al., 2020) based on gene annotation data in general feature format 3 (GFF3) format. The conserved motif of R2R3-GpMYB protein sequences was predicted using a motif-based sequence analysis tool Multiple Expectation maximizations for Motif Elicitation (MEME) version 5.1.1 program (Bailey et al., 2009). The parameters were as follows: maximum motif number of 25; other options were set to default.

Multiple sequence alignments of *G. pentaphyllum* and *A. thaliana* R2R3-MYB protein sequences were performed using molecular evolutionary genetics analysis (MEGA) version 10.1.7. Subsequently, a maximum likelihood (ML) phylogenetic tree was constructed using the FastTree version 2.1.1 (Price et al., 2009). The ML phylogenetic tree was visualized by the Interactive Tree of Life (iTOL) (Letunic and Bork, 2021). Additionally, an ML phylogenetic tree including full length of 87 R2R3-GpMYB protein sequences was constructed using the same methods. Finally, a combination of the phylogenetic tree, conserved domains, gene structures, and conserved motifs of R2R3-GpMYB protein sequences was visualized using the Tbtools software (Chen et al., 2020).

### Genomic Localization and Gene Duplication of *R2R3-GpMYB* Genes

The physical positions of the identified *R2R3-GpMYB* genes were mapped to 11 chromosomes of the *G. pentaphyllum* genome using the Tbtools software (Chen et al., 2020). The orthologous *MYB* genes between *G. pentaphyllum* and *A. thaliana* as well as those between *G. pentaphyllum* and *C. sativus* were identified using OrthoVenn2 (Xu et al., 2019). Multiple Collinearity Scan toolkit (MCScanX) was used to analyze the gene duplication events with default parameters (Wang et al., 2012). Non-synonymous (ka) and synonymous (ks) substitutions of each duplicated *R2R3-MYB* gene were calculated using the Tbtools software (Chen et al., 2020).

### Ribonucleic Acid Isolation and Quantitative Reverse Transcriptase-PCR Analysis

Total RNA isolation and qRT-PCR analysis were performed using the methods described by Xu et al. (2020). qRT-PCR was performed using the LightCycler 96 System (Roche, United States). The *GpActin* gene was used for quantitative gene expression normalization (Xu et al., 2020; Huang et al., 2021). The 2^–ΔΔ*Ct*^ analysis method was adopted to calculate the relative gene expression. Primer information is given in Supplementary Table 1.

### Yeast One-Hybrid Assays

Yeast one-hybrid (Y1H) assays were performed as described previously (Huang et al., 2018). To construct the prey vector, the open reading frame (ORF) of the *GpMYB81* gene was cloned into the pGADT7 plasmid. To construct the bait vectors, the fragments of *GpFPS1* and *GpCHS* promoters (about 1.5 Kb) were cloned into the pAbAi plasmid. Yeast cells were grown for 3 days at 30°C on synthetic dropout (SD)/-Ura/-Leu medium added with or without aureobasidin A (AbA). Primer information is given in Supplementary Table 1.

### Dual-Luciferase Assays

A dual-luciferase (LUC) reporter assay was conducted in *Nicotiana benthamiana* leaves according to the method described previously (Huang et al., 2018). To construct the effector vector, the ORF of the *GpMYB81* gene was cloned into the pK2GW7 plasmid. An empty vector of pK2GW7 was used as a negative control. To construct the reporter vectors, the fragments of *GpFPS1* and *GpCHS* promoters (about 1.5 Kb) were cloned into the pGreenII 0800-LUC plasmid. Fluorescence was detected using an *in vivo* imaging system (NightShade LB 985, Germany). Primer information is given in Supplementary Table 1.

## Results

### Identification and Characterization of *Gynostemma pentaphyllum R2R3-MYB* Family Genes

In total, 248 candidate genes were originally obtained from the *G. pentaphyllum* genome as encoding proteins that contained MYB domains. After removing the redundant transcripts, all the candidates were further verified *via* Pfam, HMMscan, and SMART. As a result, 87 *R2R3-GpMYB* genes were identified in *G. pentaphyllum*. Among these, 86 *R2R3-GpMYB* genes were mapped to 11 chromosomes and renamed from *GpMYB1* to *GpMYB86* according to their location on the chromosomes. However, one exception was observed, an *R2R3-GpMYB* gene renamed *GpMYB87* was not located on any chromosome.

The amino acid number of R2R3-GpMYB proteins ranged from 126 to 556, with theoretical isoelectric point and molecular weight values ranging from 4.97 (*GpMYB14*) to 9.87 (*GpMYB5*) and 14.68 (*GpMYB5*) to 61.53 (*GpMYB68*) kDa, respectively. To provide possible clues for functional studies, we predicted their subcellular locations. The results indicated that all the R2R3-GpMYB proteins were located in the nucleus. These results are shown in Supplementary Table 2.

### Phylogenetic Analysis and Classification of *R2R3-MYB* Genes in *Gynostemma pentaphyllum*

**FIGURE 1:**
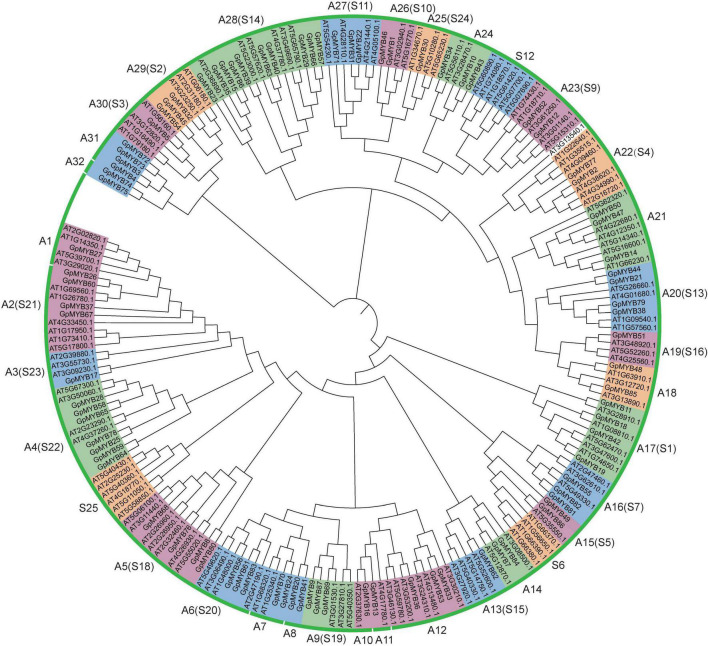
Phylogenetic tree comparison of R2R3-MYB proteins between *Gynostemma pentaphyllum* (*G. pentaphyllum*) and *Arabidopsis thaliana* (*A. thaliana*). A maximum likelihood (ML) phylogenetic tree, including full length of 87 R2R3-GpMYB and 124 R2R3-AtMYB protein sequences, was generated using the Jones–Taylor–Thornton (JTT) algorithm *via* the FastTree software. The genes of the *R2R3-GpMYB* family were divided into 32 subgroups (designated as A1–A32). In addition, the classification method of *A. thaliana* (Stracke et al., 2001) was adopted.

To elucidate the evolutionary relationship and gene function of the *R2R3-GpMYB* gene family, a ML tree containing 87 *R2R3-GpMYB* genes and 124 *R2R3-AtMYB* genes was constructed using the FastTree software (Figure 1). These 87 *R2R3-GpMYB* genes were divided into 32 subgroups (A1–A32), among which 20 subgroups (containing 61 *R2R3-GpMYB* genes) were consistent with the previously constructed phylogenetic tree of *A. thaliana* R2R3-MYB proteins. There were 10 specific subgroups in *G. pentaphyllum*, which were not clustered with *A. thaliana*. Moreover, no *R2R3-GpMYB* gene belonged to the *A. thaliana* S6, S12, or S25 subgroup, indicating that these *R2R3-GpMYB* genes may have evolved or lost in a given subgroup after divergence. The R2R3-AtMYB proteins of the same subgroup may have similar functions. For example, *R2R3-AtMYB* genes in the S6 and S12 subgroups are known to regulate anthocyanin and glucosinolate biosynthesis, respectively (Lin-Wang et al., 2010; Yu et al., 2021). Thus, these results suggested that *G. pentaphyllum* may have lost the ability to activate the accumulation of anthocyanin and glucosinolate or contained other special regulated pathways to produce these metabolites.

### Gene Structure, Conserved Domains, and Motif Composition of *Gynostemma pentaphyllum R2R3-MYB* Gene Family

**FIGURE 2:**
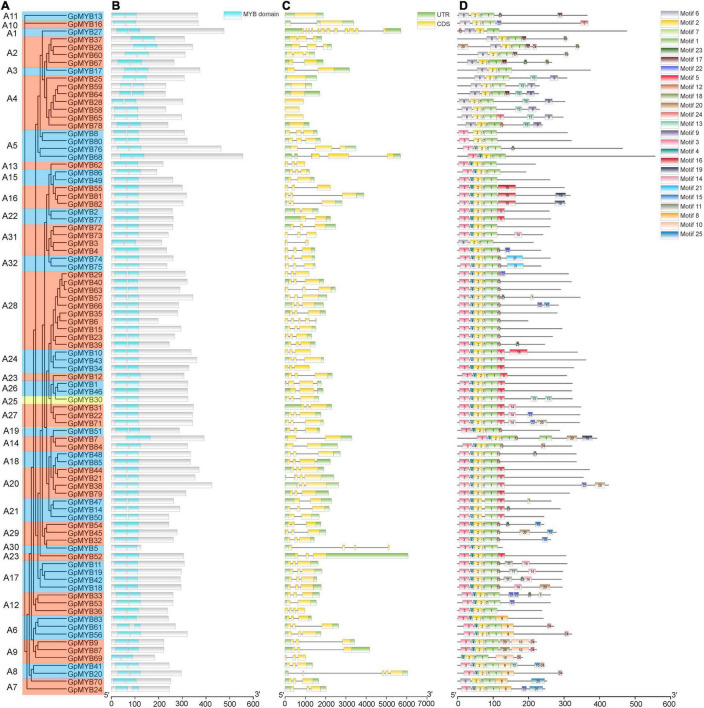
Analysis of conserved MYB DNA-binding domains, gene structure, and conserved motifs depending on the phylogenetic relationships in *R2R3-GpMYB* genes. **(A)** A phylogenetic tree was built using 87 R2R3-GpMYB proteins with the ML method. The phylogenetic tree contains 32 subgroups represented by different colors. **(B)** The conserved MYB DNA-binding domains were examined using Pfam and the Simple Modular Architecture Research Tool (SMART). Green boxes indicate conserved MYB DNA-binding domains. **(C)** Exon/intron structure analysis of *R2R3-GpMYB* genes. Gray lines, yellow boxes, and green boxes indicate introns, exons, and untranslated regions (UTRs), respectively. **(D)** Conserved motifs of *R2R3-GpMYB* genes elucidated by Multiple Expectation maximizations for Motif Elicitation (MEME). The conserved motifs are represented by the different colored boxes. The scale bar of each *R2R3-GpMYB* gene is shown at the bottom.

The typical *R2R3-MYB*-encoded proteins were characterized by R2 and R3 repeats (Dubos et al., 2010). As shown in Figures 2A,B, the 87 identified R2R3-GpMYB proteins from 32 subgroups contained two conserved MYB repeats and were separated by approximately 108 basic residues. The exon–intron structure analysis indicated that the number of exons in *R2R3-GpMYB* genes varied from 0 to 11, the majority of which contained two introns, accounting for about 65.5%. Generally, similar structures of exon/intron were observed among the genes in the same subgroup, especially the number of introns. For example, the *R2R3-GpMYB* genes in the A4 subgroup contained no intron, whereas the A17 subgroup harbored two introns (Figures 2A,C).

The conserved motifs of all the R2R3-GpMYB proteins were studied using a motif-based sequence analysis tool (Supplementary Table 3). As shown in Figure 2D, motif 1, motif 2, motif 3, motif 4, motif 6, and motif 7 in the N-terminus encoded the conserved MYB DNA-binding domain, whereas motifs in the C-terminus were highly variable. The majority of *R2R3-GpMYB* genes belonging to the same subgroup with similar functions exhibited similar motif compositions outside the MYB domain (Figures 2A,D). For example, the A2 subgroup contained motif 17 and motif 18, which played important roles in the development of axillary meristem (Lee et al., 2009), whereas motif 9 and motif 13 in the A4 subgroup participated in providing resistance to biotic and abiotic stresses (Jung et al., 2008). These results indicated that these motifs were conserved in specific subgroups, and proteins sharing these motifs within a group in the phylogenetic tree likely had similar functions.

### Chromosomal Distribution and Synteny Analyses of *Gynostemma pentaphyllum R2R3-MYB* Family

**FIGURE 3:**
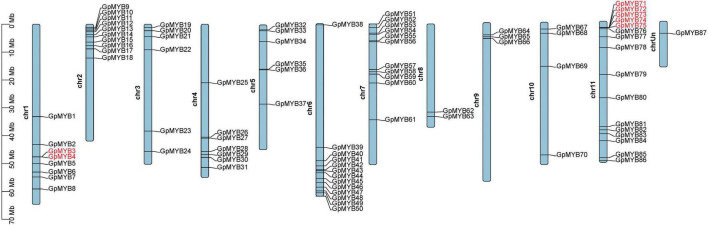
Chromosomal locations of *G. pentaphyllum R2R3-MYB* genes. Chr 1–11 represent chromosomes 1–11 and chrUn indicates an unanchored linkage group. Chromosomal locations of *R2R3-GpMYB* genes were mapped based on the *G. pentaphyllum* genome. The names of genes highlighted in red on chromosomes indicate tandem duplications.

The *G. pentaphyllum* genomic database and genome chromosomal location results revealed that 86 out of 87 *R2R3-GpMYB* genes were unevenly distributed on 11 chromosomes (Figure 3). In detail, chromosome 11 had 16 *R2R3-GpMYB* genes, accounting for the largest number of *R2R3-GpMYB* genes, followed by chromosome 7 (11 *R2R3-GpMYB* genes), whereas chromosome 8 contained only two genes and had the minimum number of *R2R3-GpMYB* genes. The majority of *R2R3-GpMYB* genes were located on both ends of the chromosome. In addition, no correlation was found between the chromosome length and the distribution of *R2R3-GpMYB* gene family members on the chromosome. According to a previous study, if two or more genes are present within 200 kb, the elements are considered a tandem repeat event (Holub, 2001). In total, six *R2R3-GpMYB* genes underwent five tandem repeat events (Supplementary Table 4).

**FIGURE 4:**
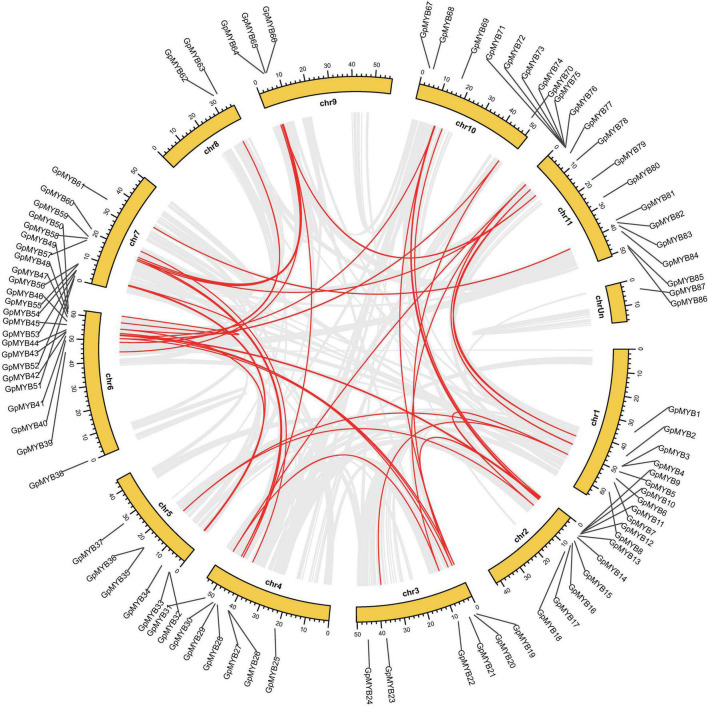
Synteny analysis of *G. pentaphyllum R2R3-MYB* genes. Chr 1–11 represent chromosome-scale scaffolds and ChrUn indicates an unanchored linkage group. All the synteny gene pairs and duplicated *MYB* gene pairs were presented by gray lines and red lines, respectively.

We employed basic local alignment search tool for proteins (BLASTP) and MCScanX to construct the collinearity of the *R2R3-MYB* gene family in *G. pentaphyllum* and identify the possible relationship and potential duplication events between them. Intrachromosomal duplications of the *R2R3-MYB* gene family were observed in the *G. pentaphyllum* genome (Supplementary Table 4). In detail, 34 pairs of *R2R3-GpMYB* genes duplicated tandemly on all the 11 chromosomes (Figure 4).

**FIGURE 5:**
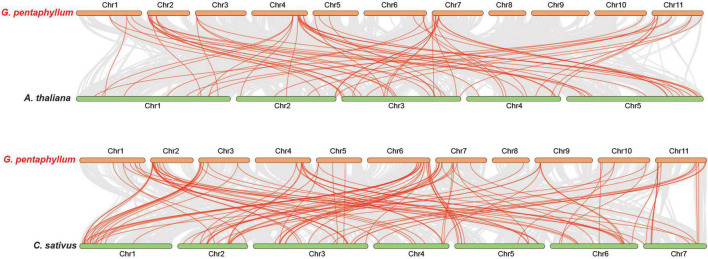
Collinearity analysis of *R2R3-MYB* genes between *G. pentaphyllum* and two representative plant species [*A. thaliana* and *Cucumis sativus* (*C. sativus*)]. Chr represents chromosome-scale scaffolds. All the gene pairs and syntenic *R2R3-MYB* gene pairs were presented by gray lines and red lines, respectively.

To further illustrate the potential evolutionary patterns of the *R2R3-GpMYB* gene family, a comparative orthologous analysis was performed between *G. pentaphyllum* and other two representative species, namely, *A. thaliana* and *Cucumis sativus* (*C. sativus*), which belong to the Brassicaceae and Cucurbitaceae families, respectively (Figure 5). The orthologous gene pairs between *G. pentaphyllum* and *A. thaliana* and *G. pentaphyllum* and *C. sativus* were 44 and 70, respectively (Supplementary Tables 5, 6). These results revealed that the identified orthologous events of *GpMYB*-*CsMYB* were considerably more than those of *GpMYB*-*AtMYB* based on the close evolutionary relationship between *G. pentaphyllum* and *C. sativus*. An extensive level of synteny conservation and increased number of orthologous events of *GpMYB*-*CsMYB* indicated that *R2R3-GpMYB* genes in *G. pentaphyllum* shared a similar structure and function with *R2R3-CsMYB* genes in *C. sativus*.

To further investigate the driving force behind the duplication of *R2R3-MYB* gene pairs in *G. pentaphyllum*, Ka/Ks (non-synonymous/synonymous substitution ratio) calculation of the duplicated *R2R3-MYB* gene pairs was performed to determine whether a selective pressure acted on the *R2R3-GpMYB* genes (Supplementary Tables 5, 6). Interestingly, all the Ka/Ks values of orthologous *R2R3-GpMYB* gene pairs were less than 1, indicating that these genes were subjected to purifying selection with limited functional divergence during evolution after duplication events.

### Identification of *R2R3-MYB* Was Related to Both Gypenoside and Flavonol Biosynthesis in *Gynostemma pentaphyllum*

**FIGURE 6:**
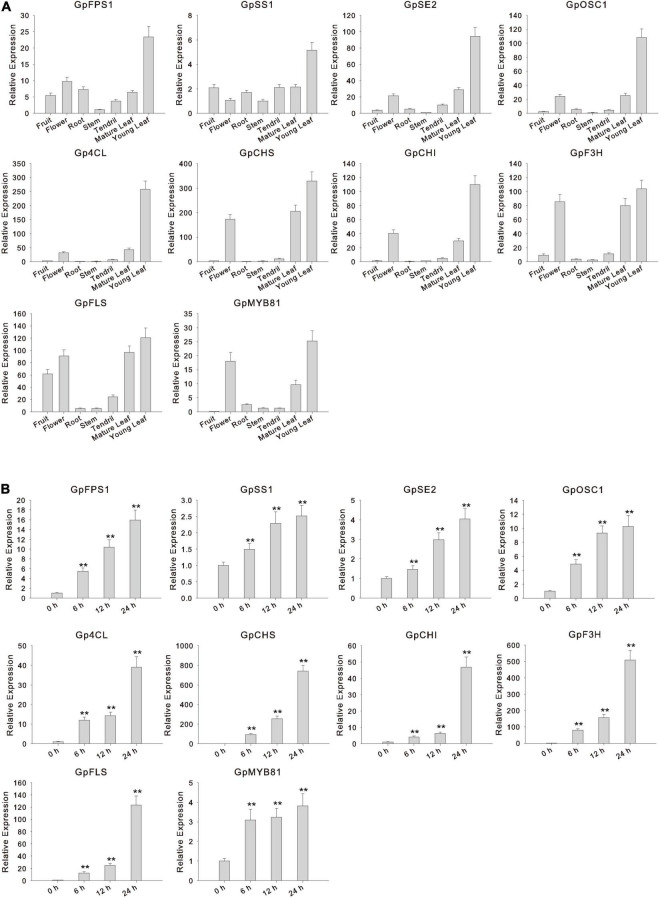
Expression profile of 10 selected candidate genes in different tissues and response to methyl jasmonate (MeJA) treatment. **(A)** Expression analysis of gypenoside and flavonol biosynthetic genes and candidate regulator *GpMYB81* in different tissues of *G. pentaphyllum*. Values represent the mean ± SE (*n* = 3 biological replicates). **(B)** Expression analysis of gypenoside and flavonol biosynthetic genes and candidate regulator *GpMYB81* with 0 (negative control), 6, 12, and 24 h treatment with MeJA. **p < 0.01 (two-tailed Student’s *t*-test). Values represent the mean ± SE (*n* = 3 biological replicates).

Transcriptional activators usually present similar expression patterns to the downstream structural genes of the metabolic pathway, narrowing the scope of screening candidate regulators and providing functional prediction. In a previous study, the early biosynthesis genes (EBGs) and late biosynthesis genes (LBGs) corresponding to gypenoside biosynthesis were elucidated (Huang et al., 2021). Furthermore, based on the gene expression profiles of different tissues (tendril, young leaf, mature leaf, root, stem, flower, and fruit), a WGCNA was performed to identify the potential upstream regulators of gypenoside biosynthetic pathway genes (Huang et al., 2021). In the constructed gypenoside biosynthesis regulatory network, five *GpMYB* genes were identified, three of which were *R2R3-MYB* genes, including *GpMYB60*, *GpMYB80*, and *GpMYB81*. Among these, *GpMYB60* was related to *AtMYB105* and *AtMYB117*, suggesting that the function of *GpMYB60* was related to the development of floral organs and the initiation of ovule outgrowth (Lee et al., 2009). *GpMYB80* was the closest homolog of *AtMYB97* and *AtMYB120*, acting as a transcriptional activator to control the differentiation of the pollen tube required for sperm release (Liang et al., 2013), and *GpMYB81* was highly close to other positive regulators of flavonol biosynthesis, such as *AtMYB11*, *AtMYB12*, and *AtMYB111* (Tan et al., 2019). In addition, flavonol biosynthetic pathway genes, including *Gynostemma pentaphyllum* 4-coumarate-CoA ligase (*Gp4CL*), *GpCHS*, *Gynostemma pentaphyllum* chalcone isomerase (*GpCHI*), *Gynostemma pentaphyllum* flavanone 3-hydroxylase (*GpF3H*), and *Gynostemma pentaphyllum* flavonol synthase (*GpFLS*), were identified in the gypenoside-related module (Supplementary Table 7). As confirmed by qRT-PCR, *GpMYB81*, along with genes encoding gypenoside and flavonol biosynthetic enzymes, showed similar expression patterns, i.e., a high expression in young leaf tissue (Figure 6A).

As an effective elicitor, MeJA can intensify the accumulation of several secondary metabolites in various medicinal plants (Zhan et al., 2018; Deng et al., 2020). Candidate genes including TFs and biosynthetic pathway genes showed similar expression patterns in response to MeJA, further narrowing down the number of candidate genes. The qRT-PCR results showed that the expression of gypenoside biosynthetic pathway genes comprising *GpFPS1*, *Gynostemma pentaphyllum* squalene synthase 1 (*GpSS1*), *Gynostemma pentaphyllum* squalene epoxidase 2 (*GpSE2*), and *Gynostemma pentaphyllum* 2,3-oxidosqualene cyclases 1 (*GpOSC1*), and flavonol biosynthetic pathway genes, including *Gp4CL*, *GpCHS*, *GpCHI*, *GpF3H*, and *GpFLS*, was significantly increased after the MeJA treatment. Moreover, among the three candidate *R2R3-GpMYB* genes, the expression of *GpMYB81* increased gradually with the increase in the MeJA treatment time (Figure 6B). According to functional predictions and co-expression patterns, we speculated that *GpMYB81* most likely functions as a “dual-function” activator of both gypenoside and flavonol biosynthesis.

### Gypenoside and Flavonol Biosynthetic Pathway Genes Were Transcriptionally Activated by GpMYB81

**FIGURE 7:**
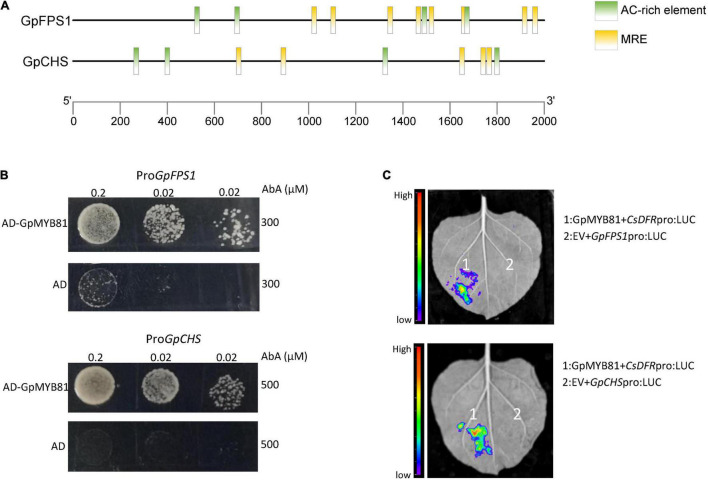
Binding of GpMYB81 to *GpFPS1* and *GpCHS* promoters and activation of their expression. **(A)** Schematic diagram of *GpFPS1* and *GpCHS* promoters. The MYB-recognition elements (MREs) and AC-rich elements, identified with manual inspection, are indicated by their respective labels. **(B)** Yeast one-hybrid assays reveal that GpMYB81 can bind to the promoters of *GpFPS1* and *GpCHS*. **(C)** Dual-luciferase (LUC) assays in *Nicotiana benthamiana* leaves were performed using *GpFPS1* and *GpCHS* promoters driving *LUC* as a reporter along with effector GpMYB81; the empty vector (EV) served as an internal control. These results showed that GpMYB81 can activate both *GpFPS1* and *GpCHS* promoters.

To investigate the mechanism underlying similar expression patterns among gypenoside, flavonol biosynthetic pathway genes, and their potential regulator GpMYB81, the transcriptional *cis*-elements of *GpFPS1* and *GpCHS* were analyzed (Figure 7A). The results revealed that the promoters of *GpFPS1* and *GpCHS* contained conserved MYB-recognition elements (MREs) or AC-rich elements, suggesting that GpMYB81 might bind to the promoters of *GpFPS1* and *GpCHS*. To prove this hypothesis, Y1H assays were performed. As shown in Figure 7B, GpMYB81 could bind to the promoters of *GpFPS1* and *GpCHS in vivo*. Moreover, the transient expression of the promoter activity assays revealed that GpMYB81 could activate the expression of *GpFPS1* and *GpCHS* (Figure 7C). These results confirmed that GpMYB81 can simultaneously activate gypenoside and flavonol biosynthetic pathway genes, thereby parallelly promoting the accumulation of gypenosides and flavonols.

## Discussion

Secondary metabolism not only endows plants with the ability to adapt to the ecological environment, but also provides abundant pharmaceutical ingredients of considerable health benefits to humans (Ramakrishna and Ravishankar, 2011; Thimmappa et al., 2014; Li et al., 2015). Terpenoid saponins and flavonoids are two important secondary metabolites, several of which have been proven to exhibit antiviral, antifungal, and anticancer properties (Zhang et al., 2018; Bonta, 2020; Russo et al., 2020; Yang et al., 2021). *G. pentaphyllum* is a traditional Chinese medicinal herb and known for its industrial use. It often serves as a natural source of gypenosides and flavonoids in medicinal products. As reported in our previous study (Huang et al., 2021), the genome sequencing of *G. pentaphyllum* was completed in 2021, providing the opportunity to perform a genome-wide analysis of the *R2R3-GpMYB* gene family. However, the *R2R3-GpMYB* gene family has not been comprehensively analyzed and their dual functionality in regulating distinct pathways of synthesis has not been well studied.

In this study, 87 members of the *R2R3-GpMYB* family were identified and characterized in *G. pentaphyllum*. Although the number of *R2R3-GpMYB* was higher than that in *C. sativus* (69) (Cheng et al., 2020), it was significantly less than that in cotton (205) (Huang J. et al., 2019), *Populus trichocarpa* (196) (Wilkins et al., 2009), and banana (285) (Pucker et al., 2020). Whole-genome duplication (WGD) is a crucial event for the rapid expansion and evolution of gene families, e.g., the recent gene duplication in several angiosperms (Pucker et al., 2020). However, WGD events and tandem duplications are rare in the *G. pentaphyllum* genome. Conversely, numerous segmental duplication events were identified in *G. pentaphyllum*, indicating that segmental duplication events were the most important factor in the expansion of the *R2R3-GpMYB* gene family, which exhibited an evolutionary pattern similar to that of *MYB* genes in other plants (Cannon et al., 2004). In addition, certain *R2R3-GpMYB* genes were not associated with syntenic gene pairs in *A. thaliana* or *C. sativus*, indicating their possible specificity to *G. pentaphyllum* during the course of evolution, providing new insight or interest to explore those special *R2R3-GpMYB* genes in the future.

Flavonoids and terpenoids, the two largest groups of specialized plant metabolites, are derived from two distinct pathways. Notably, there are several examples where MYB proteins act to regulate terpenoid and flavonoid biosynthesis. In *A. thaliana*, the R2R3-MYB TFs that are currently known to regulate flavonol biosynthesis belong to subgroup 7 (Stracke et al., 2007). Some MYB TFs involved in the regulation of triterpene saponins have also been characterized. For instance, *Panax ginseng* myeloblastosis 2 (PgMYB2) in *Panax ginseng* was characterized as a positive regulator of ginsenoside metabolism (Liu et al., 2019). However, TFs act in a coordinated manner to simultaneously regulate different pathways of specialized metabolism in a novel regulatory mechanism. In this study, *GpMYB81* belongs to subgroup 7 of the *R2R3-MYB* gene family from *A. thaliana*, which was identified in the gypenoside-related module. In addition, the expression patterns of *GpMYB81* were highly correlated with gypenoside and flavonol biosynthesis genes and with those involved in response to MeJA, further supporting the finding that *GpMYB81* can regulate the accumulation of both gypenosides and flavonols. The chalcone synthase (*CHS*) gene plays a vital role in flavonol biosynthesis and greatly impacts the content of flavonols, whereas the overexpression or RNA interference of the *FPS* gene can significantly increase or decrease the biosynthesis of gypenoside (Kim et al., 2014; Zhang et al., 2017). In this study, GpMYB81 could bind to the promoters of *GpFPS1* and *GpCHS* and increase the transcriptional activities of these promoters. These findings suggested that *GpMYB81* acts as a “dual-function” TF that regulates both gypenoside and flavonol pathways in *G. pentaphyllum*.

## Conclusion

In summary, this study presented a detailed genome-wide analysis of the *R2R3-GpMYB* gene family. A total of 87 *R2R3-GpMYB* genes were identified in *G. pentaphyllum* and divided into 32 subgroups, with an uneven distribution on 11 chromosomes. Similar exon–intron structures and conserved motif compositions of *R2R3-GpMYB* genes were observed in the same subgroup, which provided additional support for phylogenetic analysis. Synteny analysis indicated that segmental duplication events primarily contributed to the expansion of the *R2R3-GpMYB* gene family. The Ka/Ks analysis suggested that the *R2R3-GpMYB* gene family underwent purifying selection. A combination of similar gene expression patterns, Y1H, and dual-LUC assay results verified that *GpMYB81* acted as a “dual-function” activator in gypenoside and flavonol biosynthesis by directly binding to the promoters of *GpFPS1* and *GpCHS*. These results provide novel insights into the parallel transcriptional regulation of gypenoside and flavonol biosynthesis in *G. pentaphyllum*.

## Data Availability Statement

Publicly available datasets were analyzed in this study. This data can be found here: the transcriptome sequencing data can be found in NCBI under accession codes PRJNA720501 and PRJNA631355.

## Author Contributions

DH and YT conceived this project. DH and RM designed the experiments. DH and SX prepared the samples and wrote the manuscript. DH, RM, and SX analyzed the bioinformatics data. SY, LL, RH, and YT provided valuable suggestions on the research design and the improvement of the manuscript. All authors contributed to the article and approved the submitted version.

## Conflict of Interest

The authors declare that the research was conducted in the absence of any commercial or financial relationships that could be construed as a potential conflict of interest.

## Publisher’s Note

All claims expressed in this article are solely those of the authors and do not necessarily represent those of their affiliated organizations, or those of the publisher, the editors and the reviewers. Any product that may be evaluated in this article, or claim that may be made by its manufacturer, is not guaranteed or endorsed by the publisher.

## Footnotes

1
http://pfam.xfam.org

2
http://smart.embl-heidelberg.de/
